# MRI evaluation of diffuse subcutaneous neurofibroma of the lower limb in a low resource setting

**DOI:** 10.1259/bjrcr.20170090

**Published:** 2017-12-15

**Authors:** Adenike Temitayo Adeniji-Sofoluwe, Olanrewaju Adebusuyi Ogunleye, Clement Abu Okolo

**Affiliations:** 1 Department of Radiology, College of Medicine, University of Ibadan and University College Hospital, Ibadan, Nigeria; 2 Department of Radiology, University College Hospital, Ibadan, Nigeria; 3 Department of Pathology, College of Medicine, University of Ibadan and University College Hospital, Ibadan, Nigeria

## Abstract

An unusual type of neurofibroma predominantly seen in children and young adults is diffuse neurofibroma. We present a 25-year-old female with recurring soft tissue masses in her right lower limb. MRI showed areas of *T*_1_ iso-intensity and *T*_2_ hyperintensity relative to skeletal muscle within the subcutaneous fat. These masses show marked enhancement post gadolinium administration. Histological examination of the excised mass showed diffuse neurofibroma. The rare nature of this tumour and the limited literature describing the imaging features make the diagnosis relatively difficult for a radiologist.

## Clinical presentation

A 25-year-old African female presented at the MRI suite with several years history of a progressively enlarging right calf and ankle swelling. She was referred by the orthopaedic surgeon for a musculoskeletal MRI of the right calf with a clinical diagnosis of a soft tissue sarcoma. On examination, an ill-defined mass was noticed at the posterior right calf. It was warm, firm and not painful or tender to touch. A similar mass was observed at the right ankle. There was an excision of a similar swelling 10 years prior to re-presentation.

Differential diagnoses included diffuse subcutaneous neurofibroma and haemangioma.

## Investigation/Imaging

Routine haematology and blood chemical pathology tests were normal. She has no significant past medical history or family history of neurocutaneous disease.

**Figure 1.**
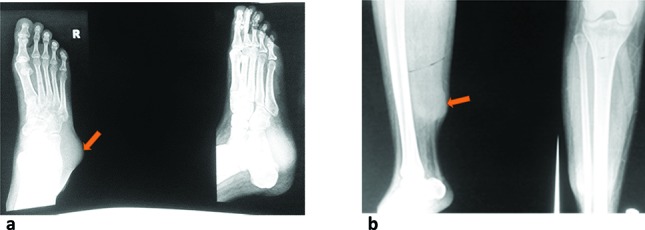
Plain radiographs showing homogenous soft tissue masses over the right ankle (a) and calf (b).

Plain radiograph (Figure 1) showed two oval well-defined homogenous soft tissue masses in the posterior right calf and at the ankle. No abnormal calcification or phleboliths were seen. The overlying skin and adjacent bones were normal.

**Figure 2.**
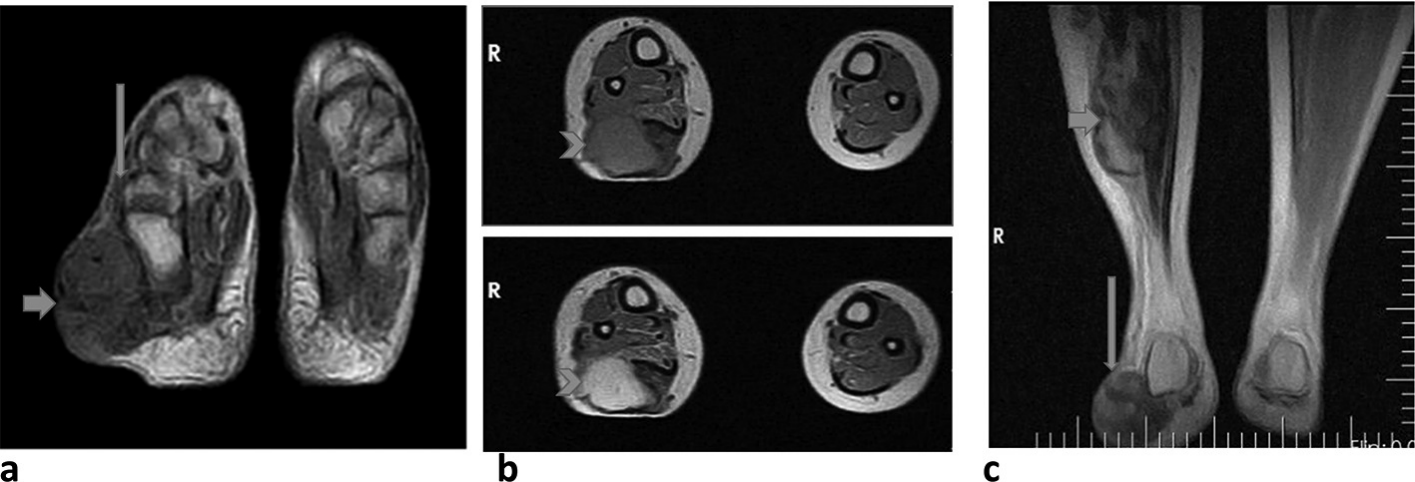
(a) Pre-contrast spin echo axial *T*_1_ weighted MR image (TR/TE, 904/16) shows a bulging mass (short arrow) in the subcutaneous tissue over the right ankle which is iso-intense to adjacent muscle (long arrow). (b) Pre- and post- contrast spin echo axial *T*_1_ weighted MR images (TR/TE, 904/16) show enhancement of the right posterolateral calf mass (arrow heads). (c) Coronal *T*_2_ weighted fat-saturated MR image (TR/TE, 2000/32) shows a bulging mass (short arrow) in the subcutaneous layer of the right calf and right ankle (long arrow) which is hyper-intense to adjacent muscle.

Serial axial and coronal *T*_1_W (*T*_1_ weighted), *T*_2_W (*T*_2_ weighted), STIR, PD and sagittal *T*_2_W as well as post contrast axial MRI images were acquired. These show a well-defined oval-shaped mass which is iso-intense on *T*_1_W and hyperintense on *T*_2_W sequences in the subcutaneous tissue of the right calf, displacing the adjacent muscles anteriorly. This mass measures 8.3 cm by 4.0 cm by 5.3 cm in longitudinal, anteroposterior and transverse dimensions, respectively. (Figure 2) A similar but smaller mass was seen in the subcutaneous tissue in the lateral aspect of the right foot. These masses show marked and bright heterogeneous enhancement after intravenous administration of paramagnetic contrast agent (Gadolinium DTPA).

**Figure 3.**
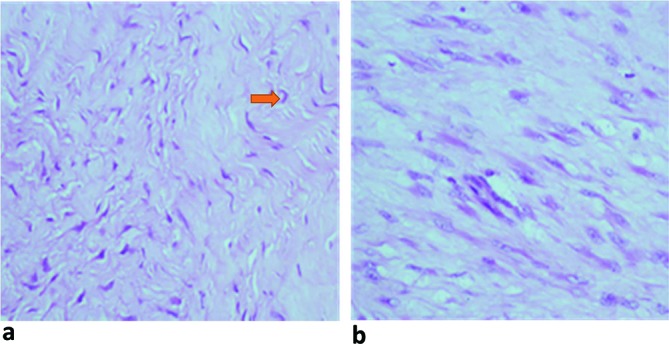
Histological features of diffuse neurofibroma. Photomicrographs of surgical specimen of diffuse neurofibroma (a, b), showing spindle shaped cells with wavy to comma-shaped nuclei (arrow on photomicrograph) on a fibromyxoid background. (a = H&E ×100, b = H&E x400).

Surgical resection of the mass was performed and subsequent histological examination reported a benign nerve sheath neoplasm composed of spindle cells disposed in sheets on a variably fibromyxoid stroma. The spindle cells have wavy to curved elongated nuclei with scant eosinophilic cytoplasm. (Figure 3).

## Treatment

Treatment of diffuse neurofibroma is by surgical resection. The index patient is doing well 3 months post surgery. Clinical recurrences may develop even after complete excision because of the infiltrative growth pattern and the multicentricity of the tumour, therefore close follow-up of patients is essential.^1–3^

## Outcome/follow-up/discussion

Neurofibromas are a group of common benign soft tissue tumours. They represent approximately 5% of all soft tissue tumours.^4^ They are divided into three histological types namely localized, plexiform and diffuse.^5^ The localized variety is the most common and most widely reported, representing approximately 90% of all neurofibroma, while the plexiform subtype is essentially pathognomonic of neurofibromatosis 1(NF1) and also is well reported.^4^ Diffuse neurofibroma is an uncommon form of neurofibroma typically seen in children and young adults.^6,7^ It has been reported in the calvarium.^8^ But it is a rare entity with a dearth in imaging literature; therefore the diagnosis may be easily missed. We present the MRI of a young adult female patient with diffuse cutaneous neurofibromatosis.

Neurofibromas are benign tumours of the peripheral nerves that develop from the proliferation of Schwann cells, perineural cells and endoneural fibroblasts.^9^ Diffuse neurofibroma is characterized by infiltration of skin or soft tissues. It grows between the subcutaneous tissues without destruction of adjacent structures.^7^ Slow growth in children with acceleration in adolescence and pregnancy is typical as seen is the patient being discussed.^7,10^ A nerve with an ovoid or fusiform mass is the usual mode of presentation of a localized neurofibroma, while plexiform neurofibroma is serpentine in appearance and more extensive. In contrast, diffuse neurofibroma is more infiltrative with extension between tissue planes with encasement of vascular and nervous structures.^10^ Diffuse neurofibroma is often poorly circumscribed extending along tissue planes.^6^ Unlike the plexiform neurofibroma which is strongly associated with neurofibromatosis1 (NF1), only an estimated 10% of patients with diffuse neurofibroma has been associated with neurofibromatosis.^1–11^ Although earlier literature suggested diffuse neurofibromas to be most frequently found in the head and neck region, more recently Hassel et al reported that the trunk and extremities are the commonest locations.^3,12^ There have been reported retroperitoneal location and associations with diffuse cystic lung disease.^13^

The characteristic MR imaging appearance of diffuse subcutaneous neurofibroma is iso or slightly hyperintense to skeletal muscle signal within subcutaneous tissue on *T*_1_W MR images and mildly or markedly hyperintense to skeletal muscle signals on *T*_2_W images. There is intense enhancement on *T*_1_W post-contrast administration.^3,4,10^ These findings correlate well with the MR findings in this case. On imaging with ultrasound and MR, localized neurofibromas are circumscribed soft tissue masses unlike diffuse neurofibromas that are less well-defined and more infiltrative.^2^ Large plexiform neurofibromas are almost always seen on a background of NF1. On MRI, although plexiform neurofibroma is mass-like, it lacks internal hypointense septations demonstrated in diffuse neurofibroma.^2^ The MR appearance and localization of diffuse neurofibroma may also be confused with those of angiomatous or fat-containing tumour, especially in a patient with a solitary lesion or one without clinical findings of neurofibromatosis.^10^ Doppler ultrasound will reliably exclude an angiomatous mass while CT can detect fat within a tumour.^14^ Plain radiographic findings of diffuse subcutaneous neurofibroma are not specific but can be used to exclude more aggressive lesions and closer differentials like soft tissue haemangioma where the presence of phleboliths is an important finding.^6^

## Learning points

1. MRI is the imaginginvestigation of choice for diffuse subcutaneous neurofibroma.
2. Other imaging modalities like plain film, ultrasound and CT can be performed to rule out differential diagnosis and to define the anatomic relationships with adjacent structures.

## Acknowledgments

We acknowledge the roles played by staff of the MRI suite and medical records unit in the Department of Radiology and Department of Pathology, University College Hospital, Ibadan, Oyo State, Nigeria for facilitating the retrieval of images, medical records and pathology slides.

## Consent

Written informed consent for the case to be published (including images, case history and data) was obtained from the patient(s) for publication of this case report, including accompanying images.

## References

[1] van Zuuren EJ, Posma AN. Diffuse neurofibroma on the lower back. J Am Acad Dermatol 2003; 48: 938–40. 12789188 10.1067/mjd.2003.141

[2] Beggs I, Gilmour HM, Davie RM. Diffuse neurofibroma of the ankle. Clin Radiol 1998; 53: 755–9. 9817094 10.1016/s0009-9260(98)80319-x

[3] de Varebeke SJ, De Schepper A, Hauben E, Declau F, Van Marck E, Van de Heyning PH. Subcutaneous diffuse neurofibroma of the neck: a case report. J Laryngol Otol 1996; 110: 182–4. 8729511 10.1017/s0022215100133122

[4] Hassell DS, Bancroft LW, Kransdorf MJ, Peterson JJ, Berquist TH, Murphey MD, et al Imaging appearance of diffuse neurofibroma. AJR Am J Roentgenol 2008; 190: 582–8. 18287425 10.2214/AJR.07.2589

[5] Coakley D, Atlas MD. Diffuse neurofibroma obstructing the external auditory meatus. J Laryngol Otol 1997; 111: 145–7. 9102440 10.1017/s0022215100136680

[6] Peh WC, Shek TW, Yip DK. Magnetic resonance imaging of subcutaneous diffuse neurofibroma. Br J Radiol 1997; 70: 1180–3. 9536912 10.1259/bjr.70.839.9536912

[7] Junewick J. Diffuse Neurofibroma. Advanced radiology services 2009; Available at: www.advancedradteaching.com/teachingfiles/93.pdf.

[8] Kumar S, Chaurasia P, Singh D, Batra VV, Aher R. Solitary giant diffuse neurofibroma of the scalp with calvarial defect. Asian J Neurosurg 2017; 12: 263–5. 28484547 10.4103/1793-5482.144199 PMC5409383

[9] Megahed M. Histopathological variants of neurofibroma. A study of 114 lesions. Am J Dermatopathol 1994; 16: 486–95. 7528474 10.1097/00000372-199410000-00003

[10] Huang G-S, Huang C-W, Lee H-S, Chang W-C, Lee C-H, Leu N-H, et al Diffuse Neurofibroma of the Arm: MR Characteristics. Am J Roentgenol 2005; 184: 1711–2. 15855151 10.2214/ajr.184.5.01841711

[11] Macias VC, Rafael M, Fernandes C, Rosa JC. Diffuse neurofibroma--an uncommon cause of alopecia. An Bras Dermatol 2013; 88: 166–9. 24346909 10.1590/abd1806-4841.20132170 PMC3876007

[12] Traoré B, Fofana Y. [The diffuse plexiform neurofibroma of the left thigh in a patient aged 78 years in dermatological environment in Bamako]. Pan Afr Med J 2017; 26: 82. 28491213 10.11604/pamj.2017.26.82.11539 PMC5409993

[13] Madhyastha SP, Gopalaswamy V, Reddy CT, Acharya RV. Interesting association of neurofibroma with diffuse cystic lung disease (NF-DLD). BMJ Case Rep 2017; 2017: bcr2016217774. 10.1136/bcr-2016-217774 PMC5278328 28122801

[14] Dubois J, Garel L, David M, Powell J. Vascular soft-tissue tumors in infancy: distinguishing features on Doppler sonography. AJR Am J Roentgenol 2002; 178: 1541–5. 12034635 10.2214/ajr.178.6.1781541

